# COVID-19 pandemic: the possible influence of the long-term ignorance about climate change

**DOI:** 10.1007/s11356-020-12167-z

**Published:** 2021-01-06

**Authors:** Shaghayegh Gorji, Ali Gorji

**Affiliations:** 1 Epilepsy Research Center, Westfälische Wilhelms-Universität Münster, Domagkstr. 11, Münster, 48149 Germany; 2 Department of Neurosurgery, Westfälische Wilhelms-Universität Münster, Münster, Germany; 3 Shefa Neuroscience Research Center, Khatam Alanbia Hospital, Tehran, Iran; 4 Neuroscience research Center, Mashhad University of Medical Sciences, Mashhad, Iran; 5 Department of Neurology with Institute of Translational Neurology, Westfälische Wilhelms-Universität Münster, Münster, Germany

## Abstract

In addressing the current COVID-19 pandemic and evaluating the measures taken by global leaders so far, it is crucial to trace back the circumstances influencing the emergence of the crisis that the world is presently facing. Could it be that the failure to act in a timely manner dates way back to when first concerns about climate change and its inevitable threat to human health came up? Multiple lines of evidence suggest that the large-scale and rapid environmental changes in the last few decades may be implicated in the emergence of COVID-19 pandemic by increasing the potential risk of the occurrence and the spread of zoonotic diseases, worsening food security, and weakening the human immune system. As we are facing progressive climatic change, a failure to act accordingly could inevitably lead to further, more frequent confrontations with newly emerging diseases.

The global mean atmospheric Co_2_ levels in the last few decades are higher than at any time in the past 800,000 years (Lüthi et al. 2008) and the last decade was the warmest decade on record at least during the past 150 years (Vitasse et al. 2018; Mann et al. 2016). There are complex and multifaceted links between human-induced climate change and global health risks (McMichael et al. 2008; Butler 2018). Climate change intensifies the risks of both direct and indirect zoonotic diseases (Bradley and Altizer 2007). A large amount of evidence suggests that the rapid ecological changes in the last few decades may be implicated in the emergence of COVID-19 pandemic by increasing the potential risk of the occurrence and the spread of infectious illnesses (Wu et al. 2016; Baylis 2017), exacerbating food insecurity (Wheeler and von Braun 2013), and weakening the human immune system (Swaminathan et al. 2014).

More than three decades ago, it was predicted that climate change could affect the prevalence, spread, and intensity of viral diseases through (i) alterations in the pathogen transmission pattern, (ii) the host’s susceptibility to infection, and (iii) the socioeconomic status (Shope 1991; Nicholls 1993; Chan et al. 1999). Climatic alterations and global warming enhance the seasonal peak and time window of future, potentially epidemic viral infections, particularly zoonotic viral diseases (Liu-Helmersson et al. 2016). Researchers in the Shanxi Province, China, (a neighboring state to Wuhan city) in 2013 have reported an association between climatic change and the alterations of transmission patterns of vector-, air-, and water-borne infectious diseases (Wei et al. 2014a; Tong et al. 2015). These studies also predicted that the elderly, children, subjects with existing chronic diseases, and outdoor workers are the most vulnerable groups to the outbreak of any climate-related zoonotic diseases (Wei et al. 2014b; Tong et al. 2015).

Furthermore, it has been suggested that climate change tends to increase the geographic expansion of infectious diseases by affecting the pathogens, vectors, hosts, and/or their living environment (Wu et al. 2016). Climate alteration has a potent impact on the replication, development, and transmission rate of viral pathogens (Ruiz et al. 2010; Ruiz-Moreno et al. 2012; Altizer et al. 2013). The ambient temperature change affects viral replication in vitro and affects the frequency of virus transmission in various in vivo models (Lowen et al. 2007; Foxman et al. 2016; Moriyama and Ichinohe 2019). Global warming was positively correlated with the rate of virus evolution in previous viral outbreaks, such as the epidemic of West Nile virus, and played a crucial role in the preservation and intensification of human infection (Paz 2015). Bats are the natural reservoir for several viruses, such as Severe Acute Respiratory Syndrome and Middle East Respiratory Syndrome viruses, which have caused the previous coronavirus outbreaks (O'Shea et al. 2014). Bats are also suspected as natural hosts for SARS-CoV-2, the causative agent of COVID-19 (Sun et al. 2020). The emerging novel coronavirus within bat communities could be due to the impact of climate change on their geographic distribution and habitat suitability in the last few years (Lorentzen et al. 2020). The implication of climate changes on habitat contracture and distributional shifts of bat populations leads to a high prevalence of coronavirus shedding and viral loads in Western Australia (Prada et al. 2019). Climate-induced mismatch in the timing of bird migration enhances viral infection prevalence and spillover potential (Brown and Rohani 2012). Stressful events, such as climate change and habitat destruction, could lead to an alteration of the immune tolerance as well as a significant enhancement of the viral replication and load of persistently infected bats, which subsequently facilitates shedding of virus (Chionh et al. 2019; Subudhi et al. 2019). The interval between the first spillover and viral outbreak originating in bats markedly decreased during the last few decades, which is probably due to several factors, such as climate change, food insecurity, and population growth (Plowright et al. 2015; Wang and Anderson 2019).

Aside from its unprecedented consequences on pathogen development and transmission, climatic alterations also strongly threaten human health by compromising food security (McMichael 2013; Wheeler and von Braun 2013; Watts et al. 2019), which plays a crucial role in human immune function, particularly on vulnerable subjects such as the elderly (Childs et al. 2019). Global warming and air pollution significantly reduce the global availability of micronutrients and vitamins (such as iron, zinc, and copper as well as vitamins C, D, and E), which can lead to the emergence of more virulent strains via changes of the genetic make-up of the viral genome (Beck and Matthews 2000; Schmidhuber and Tubiello 2007; Gorji and Khaleghi Ghadiri 2020). In addition, global warming and increased CO_2_ emissions lead to a more suitable environment for food-borne pathogens, which subsequently increase the risk of inadequate nutrient intake (Tirado et al. 2013; Myers et al. 2014; Beach et al. 2019; Macdiarmid and Whybrow 2019). The emergence of novel strains of pathogens with new pathogenic characteristics could be facilitated through enhanced mutation rates in nutrient-deprived populations (Beck et al. 1995). Furthermore, nutrient deficiencies reduce the ability to resist infections and are common causes of the immune system malfunctions (WHO 2013). Climatic alterations weaken the immune system on both individual and population levels (Swaminathan et al. 2014) and can shape the evolution of immune genes and regulate the immune gene diversity (O’Connor et al. 2020). Air pollution and greenhouse gases stimulate pro-inflammatory immune responses across different immune cells and dysregulate antiviral immune responses (Glencross et al. 2020). Nutrient deficiencies and immune system dysfunction affect the occurrence and overall outcome of viral infections (Gombart et al. 2020). For instance, multiple clinical studies indicate an association between an increased risk of viral infections and vitamin D deficiency (Laplana et al. 2018; Zhou et al. 2019). Approximately 60% of deaths from COVID-19 in Italy occurred in the Lombardy region (Statista 2020), the most air polluted areas in Italy (Conticini et al. 2020) with an extremely high prevalence of vitamin D deficiency, particularly in the cold seasons (Ferrari et al. 2019). Air pollution is associated with increased ozone and nitrogen dioxide values which lead to solar ultraviolet-B (UVB) absorption and reduce the efficiency of cutaneous synthesis, therefore facilitating vitamin D deficiency (Wacker and Holick 2013). Substantial negative correlations have been reported for associations of UVB values and population mean vitamin D levels with case fatality rate and pneumonia during the 1918–1919 influenza pandemic (Grant and Giovannucci 2009; Gorji and Khaleghi Ghadiri 2020).

In the future, more extreme climate events, such as droughts and floods, are expected to change human and wildlife behavior/migration patterns and bringing both species into a closer contact, especially during a shortage of food; an event that may have occurred in Wuhan, China, due to severe droughts in this area over the past four decades (Bell et al. 2018; Sun et al. 2020). Climate change and increasing urbanization have extensively decreased wetland areas in Wuhan, China (Wu et al. 2020), which lead to seriously restrict food production (Yu et al. 2016; Wang et al. 2020). Forcing humans to look for new food sources lead to increased sharing space with wildlife (Woods et al. 2019). Several patients who were admitted to hospitals with COVID-19 infection have links with wet animal and seafood wholesale market in Wuhan, China (Chen et al. 2020).

These observations linking climate change to numerous factors encouraging the development of novel pathogens point out that an adequate response to the current crisis is bound to involve addressing climate change. As we are facing extensive climatic changes, a failure to act accordingly could inevitably lead to further, more frequent confrontations with newly emerging infections, such as COVID-19 (Botzen et al. 2021; Kumar et al. 2021).
